# Differences in Psychological and Behavioral Changes between Children following School Closure due to COVID-19

**DOI:** 10.1155/2021/5567732

**Published:** 2021-08-21

**Authors:** Kiwamu Nakachi, Kentaro Kawabe, Rie Hosokawa, Ayumi Yoshino, Fumie Horiuchi, Shu-ichi Ueno

**Affiliations:** ^1^Department of Neuropsychiatry and Neuroscience, Ehime University Graduate School of Medicine, Toon City, Ehime 791-0295, Japan; ^2^Center for Child Health, Behavior, and Development, Ehime University Hospital, Toon City, Ehime 791-0295, Japan

## Abstract

School closure due to coronavirus disease 2019 (COVID-19) pushed children across ages and nationalities into a state of mental health crisis. In Japan, children between the ages of 6 and 18 were ordered to stay at home and observe social distancing for several months. This study is aimed at investigating the effects of quarantine due to COVID-19 on children belonging to different developmental stages in life. Data were collected from mothers of typically developing children aged between 6 and 18 years. The differences in psychological and behavioral changes following school closure during the COVID-19 pandemic were explored. A total of 535 children, including 145 students in lower grades of elementary school (6–9 years), 124 students in higher grades of elementary school (9–12 years), 132 students in junior high school (12–15 years), and 134 students in high school (15–18 years), were recruited. Children in lower grades of elementary school (lower grades group) gained significantly lower understanding about COVID-19 and the necessity of COVID-19 restrictions than children in the other groups. Moreover, they had more psychological problems: they easily cried and complained, were unable to keep calm, and were dependent on parents and family members. Changes in sleep patterns were more prevalent in junior and senior high school students. We concluded that mental health care should be provided based on the growth period of each child not only during school closure but also after school reopening.

## 1. Introduction

The coronavirus disease 2019 (COVID-19) pandemic has been affecting substantial life events worldwide. On January 30, 2020, the COVID-19 pandemic was declared a public health emergency of international concern by the World Health Organization. Measures such as hand hygiene, wearing face masks, eye protection, and maintaining person-to-person physical distance, were recommended [1]. Furthermore, schools were closed in many countries to reduce the spread of COVID-19 [2]. In Japan, elementary, junior high, and senior high school closures began on March 3, 2020, and a public health emergency of international concern was declared on April 7 by the Japanese government. Adults and children in Japan were ordered to stay at home and observe social distancing [3]. Several studies have indicated that school closure and stay-at-home orders due to COVID-19 pushed children into a state of mental health crisis and that they encountered stressors, such as fear of infection, frustration, and lack of companionship [4, 5]. Elementary school students were more afraid of the threat to life of their lives [6]. In addition, behavioral problems, such as clinginess, inattention, irritability, and anxiety [7, 8], were observed, which showed the importance of mental health care for children. School closure may have influenced Japanese children and adolescents differently based on their developmental stage.

This is the first instance of school closure for more than two months in Japan. Therefore, it is unclear how long-term school closure and quarantine affects children's mental health and behavior. To our knowledge, no study has explored the differences in psychological and behavioral changes caused by school closure and quarantine during the COVID-19 pandemic between children and adolescents in different developmental stages of life. We hypothesized that the effects of long-term quarantine are different for children as they belong to different developmental stages in life. The present study is aimed at exploring the differences in the effects of quarantine during COVID-19 in children belonging to different stages of development in life.

## 2. Materials and Methods

### 2.1. Study Design and Participants

In this cross-sectional study, we recruited participants during school closure from April 30 to May 8, 2020. We calculated the sample size before starting the recruitment process. G^∗^Power software version 3.1.9.7 was used to calculate the sample size. This study used the chi-square test. An effect size of 0.2, a significance level of *α* = 0.05, and a statistical power of 1 − *β* = 0.95 were used. Because the participants were children who were at home because schools were closed, it was difficult to access samples; therefore, snowball sampling was used to recruit participants for the study. We sent the link to four coauthors and shared the link with their acquaintances and colleagues who met the requirements. The link to the questionnaire was converted into a quick response code that was sent to mothers as a Google form via a text message. On opening the link, mothers were autodirected to information on the study, including its title, aim, and eligibility criteria. Mothers completed the questionnaire anonymously and sent their responses using electronic equipment (mobile phones, smartphones, tablets, or computers). When the recruitment period passed, we had reached our target number of participants. We did not conduct any additional recruitment. All data were protected according to the general data protection regulation. This study was approved by the Institutional Review Board of the Ehime University Graduate School of Medicine (IRB No. 2006014). The inclusion criteria were children (1) aged 6–18 years, (2) with no history of medical consultation about mental or developmental health, (3) living in Ehime prefecture, [Ehime Prefecture has a population of 1,320,000 and there were about 133,000 children aged 6-18 (10.1%) in 2020], and (4) whose mothers could fill out the informed consent form. The exclusion criteria were those (1) who did not attend elementary, junior high, or senior high school; (2) who belonged to special need classes or schools; and (3) who submitted incomplete questionnaires.

### 2.2. Questionnaire Design

The online survey included three categories: (1) demographic data, (2) questions about stress and children's understanding of COVID-19, and (3) questions about changes in children's behavior.

1. Demographic data

Demographic data were categorized based on age, sex, and level of schooling. The children were classified into four groups based on the developmental stage that they were at, referring to the current educational system of the Ministry of Education, Culture, Sports, Science, and Technology (MEXT) [9]: lower grade elementary school (grades 1–3/6–9 years of age), higher grade elementary school (grades 4–6/9–12 years of age), junior high school (grades 7–9/12–15 years of age), and senior high school (grades 10–12/15–18 years of age).

(2) Questions about stress and children's understanding of COVID-19
(3) Questions about changes in children's behavior

These yes/no questions included in the questionnaire are shown in Tables 1 and 2, respectively.

### 2.3. Statistical Analyses

The chi-squared test and Fisher's exact test were performed to explore the differences in categorical variables among the four groups. Adjusted residuals were used to compare the answers between the groups at a significance level of *p* < 0.05. The strength of the association between each categorical variable was measured using Cramer's *V*. The analyses were conducted using IBM SPSS Statistics version 22 (IBM, Armonk, NY, USA) and *R* version 3.6.3. The level of significance was set at *p* < 0.05 and adjusted residuals > 2.0 or< -2.0.

## 3. Results

### 3.1. Descriptive Characteristics

**Figure 1:**
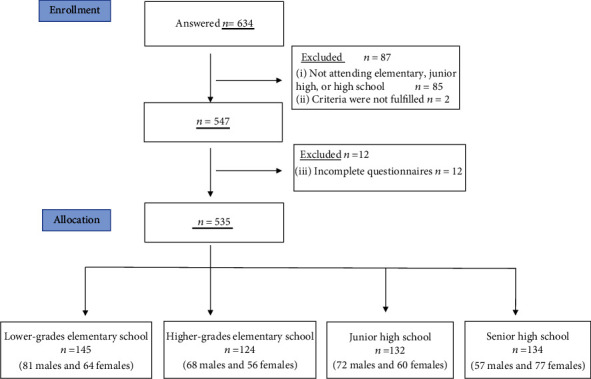
Flow diagram of the recruitment process. Figure legend: in order to study the effects of school closure, only participants attending school were enrolled. The participants were divided into four groups according to their developmental stage and grade level: lower grade elementary school, higher grade elementary school, junior high school, and senior high school.

A flowchart of the recruitment process is shown in Figure 1. We received responses from 634 participants. Answers from 85 participants who were preschool students or were not senior high school students, 2 participants from special need classes or schools, and 12 whose questionnaires were incomplete were discarded. Finally, 535 participants qualified for inclusion in the study. The average age of the participants was 11.4 ± 3.6 years. The participants included 145 children in lower grades of elementary school, 124 children in higher grades of elementary school, 132 children in junior high school, and 134 children in senior high school.

### 3.2. Between-Group Comparison of Stress and Children's Understanding of COVID-19

Table 1 shows the between-group comparison of stress and children's understanding of COVID-19 and quarantine. There were no significant differences among the four groups in the percentage of children who were stressed about the effect of COVID-19. The children in the lower grade elementary school group found it difficult to understand and follow COVID-19 restrictions (69.0%). In contrast, those in the junior and senior high school groups understood them well (94.7% and 99.3%, respectively). Moreover, children in the lower grade elementary school group did not have an adequate understanding of COVID-19 compared to those in the other three groups (89.7%). The percentage of children stressed about not being able to see their friends or teachers was lower in the lower grade elementary school group than in the other three groups (42.1%). Other variables did not significantly differ among the four groups.

### 3.3. Between-Group Comparison of Changes in Children's Behavior

Table 2 shows the between-group comparison of changes reported in children's behavior. Children spent more time at home were significantly fewer in lower grades of elementary school and significantly more in higher grades of elementary school (91.7% vs. 100%). Children in the lower and higher grade elementary school groups had significantly fewer opportunities to attend after-school activities compared to those in the junior and high school groups (84.8% vs. 89.5% vs. 70.5% vs. 67.2%). Children in the lower grade elementary school group easily cried and complained during quarantine (12.4%) and found it more difficult to keep calm (15.9%) compared to those in the other three groups. Children in the lower and higher grade elementary school groups were more dependent on parents and family than children in the junior and senior high school groups (29.7% vs. 25.8% vs. 11.4% vs. 5.2%). In contrast, children in the lower and higher grade elementary school group had significantly less change in their sleep pattern (insomnia, difficulty in falling asleep, and nightmare) than those in the junior and senior high school groups (23.4% vs. 35.5% vs. 48.5% vs. 54.5%). Other variables did not significantly differ among the four groups.

## 4. Discussion

We investigated the differences in the effects of long-term school closure during the COVID-19 pandemic in 535 children categorized based on level of schooling. To the best of our knowledge, this is the first study to explore psychological and behavioral effects of school closure during the COVID-19 pandemic in Japanese children aged between 6 and 18 years. We found that almost 80% of children across all grades were stressed about COVID-19, and that behavioral problems varied according to development stage.

Children in the lower grade elementary school group had more psychological reactions such as crying easily, complaining, difficulty in keeping calm, and dependency on parents and family than those in other groups. Although children have a lower rate of COVID-19 infection and less severe clinical manifestations compared to adults, psychological problems are more serious in them, especially in younger children [10]. In addition, we found that children in lower grades had more difficulty in understanding COVID-19 infection and the necessity of restrictions due to COVID-19 compared to older children. Previous studies have shown that lower elementary graders have a poor concept of illness. They understand that illness is transmitted from and to other people; however, they find it difficult to understand the cause of illness, such as a virus [11].

In contrast, junior and senior high school students had more physical reactions, such as changes in sleep patterns. Children belonging to these groups tend to have irregular sleep patterns during summer vacations because they were both physically and mentally inactive due to social distancing [12]. Inadequate sleep duration in children may lead to daytime sleepiness, inattention, loss of activity, and poor academic record [13]. Our results emphasize the importance of paying attention to the sleep patterns of junior and senior high school students, especially after the period of school closure.

Schools provide children with a sense of normalcy and are places where in addition to academic subjects, children learn about public health, preventing diseases, healthy exercise and eating, and orderly living. Children have decreased opportunities to learn due to school closures [14]. In addition, mental health problems in the first grade predict poor academic records in later grades [15]. Schools are also important places for providing mental health care to children. Children, especially those in lower grades, should be supported by the adults around them, including their parents, during school closure. Adults should explain about the global pandemic situation, COVID-19, and how to prevent infection in simple and easy-to-understand language. It has been reported that cartoons and animation are useful in helping children understand COVID-19 [16, 17].

Our study has several limitations. First, we were unable to use probabilistic sampling; therefore, we applied the snowball sampling method. Second, the purpose of our questionnaire was only to collect information about the children's stress and behavioral and psychological changes; therefore, we could not understand the family background, and history of developmental disabilities or mental illness in parents or siblings. In addition, there was also a lack of information about the children's background, such as their intellectual level, developmental history, mental status, and experience with counseling. Third, the answers represented the opinion of the mothers. It is necessary to create a self-administered questionnaire for future studies. Fourth, although the recruitment area of the Ehime prefecture is suburban and relatively nearly rate of the number of children aged 6-18 in Tokyo metropolis (9.6%), our findings may not be generalizable to the entire Japanese adolescent population. Fifth, because this survey was filled out anonymously, it was not possible to follow the children's progress after school reopened. Finally, the questionnaire used in this study was original, and we could not investigate the children's anxiety and sleep quality in detail. In future studies, it will be necessary to use validated scales.

## 5. Conclusions

Our survey showed that the psychological and behavioral effects of quarantine during COVID-19 vary based on developmental stages in children and adolescents. Children in low grades of elementary school had lesser knowledge about the infection and virus and had more psychological reactions than older children. Mental health care should be provided, especially for children in lower grades, not only during school closures but also after school resumes. Moreover, future studies should conduct indepth investigations of psychological and behavioral effects of school closure for more effective education of children in all grades.

## Figures and Tables

**Table 1:** Between-group comparison of stress and children's understanding of COVID-19.

Variables	Lower grade elementary school (*n* = 145)	Higher grade elementary school (*n* = 124)	Junior high school (*n* = 132)	Senior high school (*n* = 134)	*p*	Cramer's *V*
	Yes, *n* (%)	Yes, *n* (%)	Yes, *n* (%)	Yes, *n* (%)		
Has your child been stressed about COVID-19?	113 (77.9)	103 (83.1)	104 (78.8)	102 (76.1)	.573	.061
Does your child have an adequate understanding of COVID-19, such as the person-to-person transmission, unavailability of the vaccine, and isolation of infected people?	130 (89.7)^‡^	121 (97.6)	130 (98.5)	131 (97.8)	<0.001^∗∗^	.182
Does your child understand and follow COVID-19 restrictions, such as the prohibition of nonessential meetings or playing outside?	100 (69.0)^‡^	110 (88.7)	125 (94.7)^†^	133 (99.3)^†^	<0.001^∗∗^	.359
Has your child been afraid of getting infected with COVID-19?	18 (77.9)	21 (83.1)	20(78.9)	24 (76.1)	.603	.059
Has your child been stressed about the prohibition from playing outside?	97 (66.9)	88 (71.0)	82 (62.1)	79 (59.0)	.161	.098
Has your child been afraid of infecting other people?	5 (3.4)	5 (4.0)	10 (7.6)	12 (9.0)	.158	.092
Has your child been stressed about being restricted to staying with family?	6 (4.1)	8 (6.5)	11 (8.3)	17 (12.7)	.058	.118
Has your child been stressed about not being able to see friends or teachers?	61 (42.1)^‡^	75 (60.5)	74 (56.5)	75 (56.0)	.013^∗^	.142
Has your child been stressed about not being able to live in the same way as before?	52 (35.9)	48 (38.7)	50 (38.2)	56 (41.8)	.757	.047
Has your child been stressed about wearing masks?	32 (22.1)	27 (21.8)	18 (13.7)	22 (16.4)	.199	.093
Has your child been stressed about washing hands?	8 (5.5)	10 (8.1)	5 (3.8)	3 (2.2)	.159	.098
Has your child been stressed about being prohibited from visiting crowded places?	48 (33.1)	37 (29.8)	29 (22.1)	36 (26.9)	.208	.092
Has your child been stressed about school closures?	70 (48.3)	77 (62.1)	78 (59.5)	75 (56.0)	.117	.105

The chi-squared test and Fisher's exact test were used for statistical analysis. COVID-19: coronavirus disease 2019. *p* < 0.05^∗^, *p* < 0.001^∗∗^, adjusted residuals > 2.0^†^, adjusted residuals < −2.0^‡^.

**Table 2:** Between-group comparison of changes in children's behavior.

Variables	Lower grade elementary school (*n* = 145)	Higher grade elementary school (*n* = 124)	Junior high school (*n* = 132)	Senior high school (*n* = 134)	*p*	Cramer's *V*
	Yes, *n* (%)	Yes, *n* (%)	Yes, *n* (%)	Yes, *n* (%)		
Has your child spent more time at home since schools were closed?	133 (91.7)^‡^	124 (100)^†^	130 (98.5)	132 (98.5)	<0.001^∗∗^	.143
Has your child spent more time studying since schools were closed?	56 (38.6)	46 (37.1)	65 (49.2)	62 (46.3)	.134	.112
Has your child spent more time exercising since schools were closed?	25 (17.2)	16 (12.9)	11 (8.3)	15 (11.2)	.151	.094
Has your child had fewer opportunities to attend after-school activities?	123 (84.8)^†^	111 (89.5)^†^	93 (70.5)^‡^	90 (67.2)^‡^	<0.001^∗∗^	.225
Has your child been confused by the change in their schedule and daily routine due to COVID-19?	35 (24.1)	39 (31.5)	45 (34.1)	43 (32.1)	.285	.084
Has your child cried and complained easily during the quarantine?	18 (12.4)^†^	6 (4.8)	1 (0.76)^‡^	1 (0.75)^‡^	<0.001^∗∗^	.227
Has your child gotten angry and annoyed easily?	52 (35.9)	39 (31.5)	40 (30.3)	31 (23.1)	.141	.101
Does your child have decreased motivation?	51 (35.2)	45 (36.3)	60 (45.5)	62 (46.3)	.122	.104
Has your child's ability to keep calm decreased?	23 (15.9)^†^	7 (5.6)	6 (4.5)	6 (4.5)	<0.001^∗∗^	.182
Is your child excessively scared?	5 (3.4)	1 (0.8)	1 (0.8)	1 (0.7)	.161	.098
Has your child's dependence on parents and family increased?	43 (29.7)^†^	32 (25.8)^†^	15 (11.4)^‡^	7 (5.2)^‡^	<0.001^∗∗^	.263
Have your child's eating habits changed (e.g., overeating and refusing food)?	24 (16.6)	30 (24.2)	27 (20.5)	27 (20.1)	.488	.067
Has your child's sleep pattern changed (e.g., insomnia, difficulty falling asleep, and nightmares)?	34 (23.4)^‡^	44 (35.5)	64 (48.5)^†^	73 (54.5)^†^	<0.001^∗∗^	.249

The chi-squared test and Fisher's exact test were used for statistical analysis. COVID-19: coronavirus disease 2019. *p* < 0.05^∗^, *p* < 0.001^∗∗^, adjusted residuals > 2.0^†^, adjusted residuals < −2.0^‡^.

## Data Availability

The data in this study are not publicly available. The study participants were informed that the data used in this study will not be provided to third parties other than the researchers and the participants in accordance with the protocol.
